# Editorial: Emerging mechanisms of host-pathogen interactions and immune responses

**DOI:** 10.3389/fcimb.2025.1730702

**Published:** 2025-11-12

**Authors:** Aabid Hussain, Haris Saeed, Sehbanul Islam, Priyadarshi Soumyaranjan Sahu, Arpit Kumar Shrivastava

**Affiliations:** 1 Department of Genomic Sciences and Systems Biology, Cleveland Clinic Research, Cleveland Clinic, Cleveland, OH, United States; 2 Department of Medicine, Keck School of Medicine, University of Southern California, Los Angeles, CA, United States; 3 Cancer Biology Division, Perelman School of Medicine, University of Pennsylvania, Philadelphia, PA, United States; 4 Microbiology & Immunology, Medical University of the Americas, Charlestown, Nevis, Saint Kitts and Nevis; 5 Office of International Relations, Indian Institute of Technology Indore, Indore, MP, India

**Keywords:** virulence factors, molecular mimicry, adaptive and acquired immunity, microbiome, host defense, apoptosis, ferroptosis, autophagy

## Introduction

Host-pathogen interactions are dynamic and multifaceted processes where the host detects and deploys innate and acquired immune responses to eliminate the pathogens. In contrast, pathogens employ strategies to infect, evade, and manipulate the host defenses (Finlay and McFadden, 2006). Host defenses activate innate immune sensors, such as inflammasomes, toll-like receptors (TLRs), and other pattern recognition receptors (PRRs), alongside adaptive responses to identify pathogens and trigger inflammation (Kawai and Akira, 2010). However, many pathogens have developed strategies to suppress or escape these responses, highlighting the ongoing nature of the immunological arms race. These strategies include the subversion of autophagy alongside host responses mediated by innate immune sensors, molecular mimicry, and the release of virulence factors (Pradel et al., 2020). Microbial proteins activate signaling pathways that induce or inhibit apoptosis, contributing to disease pathogenesis (Häcker, 2018). Recent findings showed that non-coding RNAs, microbiome, epigenetic, and metabolic reprogramming influence host-pathogen interactions by regulating immune responses (Maguire et al., 2024; Fardi et al., 2023; Pepke et al., 2024; Malla et al., 2025; Lv et al., 2025; Darweesh et al., 2025). Advances in high-resolution imaging and spatial transcriptomics have unveiled the molecular interactions within tissue microenvironments, shedding light on immunological heterogeneity and infection circumstances (Chattopadhyay et al., 2018). These insights will help to develop precision therapies, including immunotherapies and vaccines, to enhance infectious disease control.

This editorial summarizes twelve review and research articles published in the Research Topic “Emerging Mechanisms of Host-Pathogen Interactions and Immune Responses” that provide insights into microbiome-host crosstalk, immune system dynamics, infectious disease mechanisms, and their therapeutic implications. Several studies provided insights into the cell death mechanisms, such as apoptosis, in dengue virus-induced pathogenesis. Firstly, Niranjan et al., revealed that dengue virus type-2 (DENV-2) infection results in the upregulation of matrix metalloproteinases (MMPs) such as MMP-2, MMP-9, MMP-14 and VEGF in THP-1 cells, primary monocytes and mice, In addition, a study showed that DENV-2 infection trigger apoptosis and monocyte-mediated angiogenesis, which may lead to endothelial dysfunctions similar to the mechanism of dengue shock syndrome pathogenesis. These DENV-2–mediated effects are reversed by atorvastatin, which offers protective benefits against dengue virus disease (Niranjan et al.). Another study investigated the effect of MMP-2, which is secreted by NS1-activated neutrophil, on lung epithelial cells and unveiled that it induces cell death by upregulating the expression of pro-apoptotic genes. In addition, atorvastatin diminished the release of MMP-2 and subsequently reduced the apoptosis. They suggest that the interaction of NS1-activated neutrophils with the alveolar epithelial cells participates in the lung pathogenesis involved in ARDS in severe dengue disease (Niranjan et al.). In addition, Chelluboina et al., showed that maternally acquired neutralizing antibodies against dengue infection may be protective up to six months in infants and capable of promoting dengue viral infections upon re-exposure in the later stage of life (Chelluboina et al.). Moreover, a study revealed the role of another cell death mechanism, ferroptosis, in the *Babesia microti*-mouse-*Haemaphysalis longicornis* infection model. In vector-borne diseases, revealing a novel vector-pathogen-host interaction, and a tick ferroptosis pathway facilitates *Babesia microti* acquisition. Infected ticks showed downregulation of histamine-releasing factor (HRF), ferritin 1, and GPX4, which results in elevated midgut iron and ROS levels, leading to ferroptosis. *In vivo* studies show that B. microti load increased by the ferroptosis promoter Erastin, while it decreased by the inhibitor Ferrostatin-1. Overall, the study reveals a unique mechanism by which *B. microti* manipulates ferroptosis in ticks to establish disease, providing novel insights into Babesia and tick interactions, and controlling tick-borne diseases (Chen et al.).

The Research Topic includes other complementary studies that emphasize the importance of diverse types of immune cells in shaping infectious disease outcomes. In this context, Shoeran and Anand revealed the complex interplay between autophagy and macrophage polarization in inflammation and infection conditions (Shoeran and Anand). Wang et al. showed that the neutrophil-to-lymphocyte ratio (NLR) is a significant predictor of post-trauma nosocomial infections in healthy populations. NLR ratio varies by sex and age, with a broader reference range in older adults, particularly females. Although the NLR ratio cannot pinpoint pathogens and infection sites, it may be a reference for identifying fungal infections (Wang et al.). Furthermore, a study enhances the comprehension of the CD103 marker beyond its established function in T and dendritic cells and offers novel insights into its regulation by macrophages. Normally, CD103 is expressed at low levels but is elevated in M-CSF-derived macrophages stimulated with TLR agonists through the p38 MAPK pathway. In conclusion, they demonstrated that macrophages may also produce CD103, challenging the idea that CD103 is only found in T and dendritic cells (Bouzeineddine et al.).

Host–microbiota relationships also emerged as a key research theme of this Research Topic. Notably, a review article summarizes the studies about the intricate balance between mucosal immunity and the gut microbiota in ulcerative colitis progression, providing a reference for further clinical treatment of this patient population, and discussing potential therapeutic applications (Bu et al.). In addition, a review article examined the critical components of the human microbiota-associated models, such as donor selection criteria, fecal sample collection and processing, recipient animal preparation, and fecal microbiota transplantation. They suggest that human microbiota is critical for human disease associations in mouse models, enabling translational research (Huang et al.).

This Research Topic also contains a comprehensive review that explores the underlying molecular mechanisms governing cGAS-STING signaling and its role in host immune response to pulmonary infections (Xu and Zhu). Another review article, Yin et al. focused on viral immune evasion mechanisms, which pose a significant challenge to vaccine progress. Enteroviruses inhibit host immunity by obstructing PRRs such as TLRs and RIG-1-like receptors (RLRs), by interfering with nuclear factor kappa-B (NF-κB) and Janus kinase/signal transducers and activators of transcription (JAK–STAT) pathways (Yin et al.). Lastly, Jespersen et al., associate oral diseases with the impaired cytokine responses in patients with cervical necrotizing soft tissue infection (NSTI) or cerebral abscess (CA). The decreased cytokine production after stimulation of blood cells from previous cervical NSTI or CA patients, with LPS and Poly I: C, indicates a reduced anti-bacterial and anti-viral proinflammatory response. In addition, the clinical dental examinations found a high prevalence of oral pathologic conditions (Jespersen et al.).

The research article and reviews compiled in this Research Topic underscore the host–pathogen relationship’s adaptive nature and complexity. The review articles summarize the major milestones achieved in understanding the host-microbe interaction in infectious diseases. On the other hand, the research articles identified a clear picture of how pathogens interact with the host immunity to influence the disease outcomes and identified specific pathways that can be harnessed for therapeutic purposes. For instance, cellular pathways such as apoptosis, ferroptosis, autophagy, and immune signaling mechanisms have been shown to play essential roles during infection. Although targeting autophagy presents a promising therapeutic avenue to combat infectious diseases, challenges related to host tissue damage, immune modulation, and pathogen adaptation remain unresolved. It is therefore worth exploring whether pathogen-derived factors that alter inflammatory pathways may cooperate to influence host autophagy regulatory genes, potentially contributing to neuronal damage during infection (Sahu and Ter, 2018).

Further research is needed to identify host factors essential for the pathogenesis of infectious diseases, which can be systematically uncovered using genome-wide CRISPR-Cas9 screens (Binnie et al., 2021). Complementary approaches, such as single-cell RNA-seq and quantitative proteomics, can identify key players involved in infectious diseases. These analyses can also reveal genes, signaling networks, and pathways such as apoptosis and autophagy that are manipulated by a particular pathogen (Gong et al., 2024). In addition, high-throughput datasets on host–pathogen interactions, compiled in public databases, provide a valuable resource for understanding infection biology (Le et al., 2022). Altogether, integrating multi-omics, systems biology, and immunogenomics approaches can bridge critical knowledge gaps and advance next-generation precision medicine, immunotherapies, and vaccines.

The virulence factors can be pharmacologically targeted to make it less virulent and cleared through the host’s immune system. Similarly, the host immune system can be boosted through host-directed therapies to make it effective against microbes (Munguia and Nizet, 2017). Researchers have discovered natural agents as immunomodulators isolated from mushrooms that have shown significant therapeutic potential against infectious diseases (Hussain et al., 2020; Xu et al., 2023; Wang et al., 2023). The emergence of multidrug-resistant pathogens and the scarcity of novel antibiotics pose significant challenges to the effective treatment of infections. In addition, sessile pathogenic infections that develop biofilms are usually resistant to antibiotics and the immune system. Various strategies, including antibacterial mimics such as metal complexes of TiO2, Fe(III), Mg(II), and Cu(II), have been shown to possess antibacterial and antibiofilm properties. (Khan et al., 2020; Ahmad et al., 2021; Lee et al., 2024). Moreover, microbial proteins or host factors critical for infection can be selectively eliminated through targeted protein degradation using PROTACs. These are the small molecules that recruit E3 ligases to target proteins and promote their proteasomal degradation (Espinoza-Chávez et al., 2022; Islam et al., 2024). Among the approximately 600 E3 ligases encoded in the human genome, only a few, such as CRBN, VHL, KEAP1, cIAP, and MDM2, have been successfully utilized for the development of PROTAC molecules (Bricelj et al., 2021; Barik et al., 2023; Islam et al., 2025). In conclusion, a deeper understanding of host-pathogen interactions will help to develop novel therapeutics for infectious diseases.
